# On Delirium Ebriositatis

**Published:** 1823-11

**Authors:** And. Blake

**Affiliations:** Surgeon, 5th Regiment.


THE LONDON

### Medical and Physical Journal.

5 OF VOL. L.] NOVEMBER, 1823. [No. 297.

For many fortunate discoveries in medicine, and for the detection of numerous errors, the world is indebted to the rapid circulation of Monthly Journals; and there never existed any work, to which the Faculty, in Europe and America, were under deeper obligations, than to the Medical and Physical Journal of London, now forming a long, but an invaluable, series.—RUSH.

### ORIGINAL COMMUNICATIONS,
### SELECT OBSERVATIONS, &c.

ART. I.—*On Delirium Ebriositatis.* By AND. BLAKE, Esq. Surgeon, 5th Regiment.

THE affection described by Drs. Pearson and Armstrong under the denomination of brain fever, and by Dr. Sutton, late physician to the Forces, under that of delirium tremens, I would call Delirium Ebriositatis, and define as follows:—Indirect debility of the nervous energy, succeeded by a morbid increase of action in the brain and nerves, attended with delirium, and terminating either in profound sleep or in effusion on the brain.

This is a disease peculiarly prevalent amongst the lower order of Europeans resident within the Tropics, and hence becomes a subject well worthy of the attention of the military medical officers obliged to serve in such latitudes. As the predisposing cause of the complaint is admitted, by all authors on the subject, to be the habitual abuse of diffusible stimuli, but more particularly of ardent spirit, it will, of course, occur in every part of the world where such customs prevail, and in the direct ratio of the facility with which such articles are attainable. The West Indies, where rum is an object of such little value, and where the debilitating effects of elevation of temperature excite the most temperate to allay their thirst, and thus lead insensibly to the acquirement of such habits, will appear a very likely residence to furnish frequent examples of this curious and alarming malady; and it will generally be met with in regiments in proportion to the length of their residence in these islands. I have had frequent opportunities of observing its progress and effects, particularly within the last year; and shall now relate the result of the observations I have made on this subject. The authors already quoted, the only persons who, to my knowledge, have written on the disease, appear all to coincide, with but little variation, in the nature of its cause and the *methodus medendi;* but they do not seem, *to me*, to have sufficiently explained its different stages and phenomena, and have omitted some of its pathological characters, as well as some essential points in the treatment of this formidable complaint. From all the observations I have made, and more particularly from the table which I shall here subjoin of the duration of the various symptoms of the cases which presented themselves in the right wing of the 5th Regiment within the year, I am induced, for the better description of the disease, to divide it into three distinct stages; and the regularity with which they have occurred in every case, makes me consider it as a disease *sui generis*, somewhat similar in its progress to a paroxysm of ague; to which, indeed, it bears no small analogy, being to the brain and nerves what intermittent fever is to the arterial system. In the ten cases alluded to, the first stage, or that of nervous exhaustion, came on within five days from the date of admission into hospital, which may be regarded also as the date of abstaining from spirituous liquors: it succeeded to trivial affections of various sorts, such as slight febrile action, gastric derangement, &c. and in one instance to ambustio. These indispositions were always the immediate effect of excesses.

The first indications of the disease were usually as follow:— Slowness of the pulse, frequently as low as sixty in a minute; coldness of the hands and feet, which were commonly bedewed with a clammy, icy moisture, and accompanied with general debility and diminution of temperature, owing, of course, to the defect of sensorial or nervous influence; cramp in the muscles of the extremities, with nausea and occasional vomiting, were also troublesome; the bowels were generally open, but sometimes the contrary; nervous tremor of the hands and tongue, the latter being moist and but slightly furred, formed also prominent features in this stage. All these were accompanied with dejection of spirits, frequent sighing, and oppression at the præcordia, anxiety and depression of countenance, and short and interrupted slumbers, with alarming and frightful dreams.

As the second stage approached, the countenance gradually assumed a wild aspect; the patient became restless, and a peculiar quickness was observable in his answers, with an apparent anxiety to perform immediately whatever you desired, or even to anticipate you in what he thought you were about to require. I have been generally able to prognosticate approaching delirium by this last symptom. The duration of this stage will be invariably in proportion to the nature and extent of the cause, and the state of the constitution and previous habits of the patient. Thus, in a young and healthy subject, where habits of excess have not been of long standing, and consequently where the resources of nature have not been much exhausted, nervous re-action, or the stage of excitement, will come on much quicker than in those persons whose systems have been worn out by the repeated and destructive application of spirits to the stomach, and *vice versa:* it seldom, however, under any circumstances, lasts more than a few hours.

When the second stage is established, a train of symptoms consonant with high nervous irritation follows: mental alienation, in various degrees and forms, is developed; and with this exertion of the nervous power to re-establish the state of energy, or rather excitement, which existed previous to the cessation of the application of diffusible stimuli to the nervous system through the medium of the stomach, the heart and arteries sympathise; the pulse becomes quicker, though it continues small, and the heat of the surface increases: there is, however, throughout the disease, a marked difference between the temperature of the hands and feet and the rest of the body; the former retaining, in some degree, the icy and clammy feel already spoken of, while the rest of the surface may become hot and dry. If this state continues long without amelioration, a clammy sweat pours from the skin, accompanied with high nervous irritability; the disorder of the mind increases; and objects of the most frightful forms present themselves to the imagination of the patient, and in positions in which, as Dr. Pearson says, it is physically impossible they can be situated. I recollect having seen a very distressing instance of this sort. The unfortunate sufferer, for a considerable time before his death, imagined he saw the devil at the ceiling above his bed; and, as the disease, which terminated rapidly, increased, he fancied the evil spirit approached him with a knife to cut his throat, and actually expired, making violent exertions to avoid the fatal instrument. Fortunately, however, the disease does not always assume so violent a form, and, when judiciously treated, will, in the greater proportion, terminate favourably. The mental bias is generally of the melancholic sort, usually concerning some misfortune to which the patient was liable previous to his illness: thus, a soldier will often think he is accused of some military crime, and his whole attention will be occupied in endeavouring to exculpate himself of it. If he has been much on service, he perhaps fancies he sees the enemy coming in at the window with fixed bayonets; and, if he has been at any time of his life religiously disposed, he supposes he has committed some deadly sin, for which he cannot expect forgiveness, and calls frequently on his comrades to read prayers to him, as he is soon to appear before his God. During all this he makes frequent endeavours to get out of bed, and cannot be convinced of the fallacy of his ideas. At the same time he is generally tractable, if properly managed, and will, in most instances, attend for an instant to what his medical attendant advises, and will even answer questions rationally; but he almost immediately relapses into his erroneous train of thought. From the moment delirium is fairly established, the patient is deprived of the restoring solace of "balmy sleep," and is harrassed by obstinate pervigilium, which may be looked upon as a pathognomonic symptom of the second stage of this disease. During this state the countenance is particularly anxious; the tremor of the hands and tongue continue, and the latter becomes more furred; the urine is scanty and pale, and the bowels rather confined; or, if relaxed, the stools are dark-coloured. The pupils are contracted, but there is no intolerance of light. When these symptoms have continued for one or two, or even three days, where a fatal termination is not about to take place, I have always observed a gradual mitigation of them, accompanied with a strong tendency to sleep, exhibited by yawning and drowsiness, which, as soon as it supervened, in general became profound, and lasted from six to eighteen hours, and occasionally longer, and constituted the third stage of this nervous paroxysm, or general relaxation of the nervous action, similar to capillary relaxation of the arterial system in the sweating stage of ague; and to which, in every instance that I have seen, convalescence has regularly succeeded: but, where the third, or sleeping stage, does not occur, the general symptoms increase in violence; the mind appears to labour under excessive irritation; and the patient makes violent struggles, attended with copious perspiration, which, as the disease advances, becomes deadly cold; the pulse increases in rapidity, becomes thready, and declines in strength; the tremor of the hands extends to the whole frame, and approaches to subsultus tendinum, though it does not exactly resemble that affection; the pupils become exceedingly contracted, the countenance pale and anxious, the tongue brown and dry in the centre; the patient talks incessantly, and with astonishing rapidity; the delirium becomes excessive, and continues till a short time before death. In one case, at this stage, the mind was so diseased, that, after having desired the patient to put out his tongue, he continued for nearly half an hour drawing it in and putting it out alternately, in quick succession, whenever I looked towards him, from a morbid association of ideas. There is generally, however, a calm previous to death, which in most instances takes place without a struggle. During the course of this disease, the patient seldom complains of any local pain, and, if you ask him how he feels, will answer that he is very well.

I have thus briefly described the three stages which have presented themselves to my observation in this disease. The first, or stage of exhaustion, we may compare to the cold stage of ague. The second, or that of nervous excitement, to the hot fit; and the sleeping, or third stage of this disease, to the sweating stage of intermittent fever.

The predisposing cause of this affection is agreed upon, by all authors, to be the habitual and excessive abuse of ardent spirits; though this predisposition may be induced by the immoderate and long-continued use of any of the diffusible stimuli.

The exciting cause appears to me to be the sudden cessation of the application of such stimuli to the nervous system, through the medium of the digestive organs; the powers of which sink, in consequence, to the lowest ebb; and, in endeavouring to rally and re-establish the equilibrium between the nervous and vascular systems, its efforts exceed the debilitated faculties of the sensorium, and thus excite delirium. It appears immaterial under what form the immediate excitement follows the predisposing cause; the complaint, when it occurs, is always the same. When this disease terminates fatally, it does not seem to me to be owing to venous congestions, as Dr. Armstrong asserts in his valuable work: I would rather ascribe it to serous effusion within the cranium. We are told by Baron Larrey, that, "in paroxysms of rage, and in all violent passions of the mind, the function of absorption appears to be suspended, and, consequently, an accumulation of fluids obtains at these times in the serous cavities." The irritation which exists during violent delirium must be equivalent to violent passions, and consequently produce the same effect. Dissection, as far as my experience goes, bears me out in this opinion. The fatal case which occurred during this year afforded ample proof of my assertion. The result of the examination was as follows:—(the report is drawn up by Mr. Home, hospital-assistant to the Forces, who aided me in the post-mortem examination.)

On bringing the surface of the brain into view, it did not exhibit any marks of recent inflammatory action; and, with the exception of a small quantity of coagulable lymph, which, on removing the dura mater, was found thrown out between that coat and the tunica arachnoidea, appeared otherwise healthy. All the ventricles contained a considerable quantity of serous fluid, but more especially the two lateral, which were very much distended. The choroid plexi showed no marks of turgescence. The contents of the thorax and abdomen assumed a natural appearance. The liver was small-sized, but healthy in its parenchyma.

The diagnosis in this disease is not difficult, if attention be paid to the symptoms already detailed, and which do not resemble those of any other disorder: there may, however, it is thought, be some little doubt on the minds of those unaccustomed to meet with cases of this kind, in distinguishing it from mania, Dr. Armstrong mentions two cases which assumed the character of confirmed madness; in consequence of which he says, "there can be no question but this disorder may identify itself with the true mania in peculiar subjects." As a mark of distinction in this species of alienation, it is said that the hallucination is solely concerning the patient's private affairs; but this I have not always observed to be the case, though it generally is so: besides, it is alleged that in mania the mental aberrations may be on the same subject. Perhaps, we should arrive nearer a pathognomonic distinction in stating that, in cases of mania, the mental derangement increases at the appearance of day-light; while the contrary is the case in the disease in question,—all the symptoms becoming more violent at night, and undergoing a sort of remission as the day begins to break. By this it would appear that confirmed madness is beyond the powers of febrile revolution, while this disease is still within its precincts.

Some difficulty will be experienced in forming a just prognosis in this disorder, it being so very insidious in its effects. In giving it, we must, of course, be guided by the constitution and habits of the patient, as well as by the violence of the symptoms with which he is afflicted. In worn-out and diseased habits, much addicted to the abuse of spirituous liquors, more is to be feared; as in these cases the indirect debility, or exhaustion, may be such as to require an effort too great for their worn-out nervous system to support, in its endeavours to restore order: on the contrary, in young and healthy subjects, this disease is seldom fatal, when properly treated; but, in either cases, we shall be considerably assisted in our opinion by attention to the favourable or unfavourable symptoms detailed in the commencement of this paper. The state of the pulse has always been to me a very sure guide; as, when its frequency did not exceed one hundred strokes in a minute, I looked on the patient as safe: on the contrary, when, from its rapidity and the tremor of the hands, it could scarcely be counted, I considered him in imminent danger.

In the method of cure, I would recommend that particular attention should be paid to the various stages of this complaint; and, as in each a different series of symptoms exists, so I would suggest a deviation in the mode of treatment. The age, temperament, habits, and integrity of the constitution of the patient, become also necessary objects for our consideration. Very great care should likewise be taken to soothe all irritation of mind, by every moral as well as physical means. During the first stage much may be done, the disease cut short, and a prevention of the second stage attained, under proper management. If we are fortunate enough to see the case before delirium has set in, we ought to prescribe according to the symptoms: thus, if slight gastric derangement is present, with nausea and occasional vomiting, I have found effervescent draughts, in which there were ten drops of laudanum, administered every second hour, with emollient, and (if necessary) anodyne, enemata, very efficacious. In the intermediate hours I have been in the habit of giving an ounce of rum, with a little warm water and sugar; and of prescribing the warm bath, or tepid affusion, or even the cold affusion, according to the strength of the patient, and probability of the production of arterial re-action: thus, in a young and healthy subject, I would have recourse to the cold affusion, and *vice versa.* If the warm bath should be preferred, it ought not to be of a temperature high enough to induce indirect debility. I would also recommend anodyne frictions to the epigastrium; and that the head should be shaved, and well rubbed with strong volatile liniment, so as gently to stimulate the surface of the scalp. In the event of the stomach being retentive, and not at all affected by nausea, I gave an ounce and a half of camphor mixture, with twenty or thirty drops of æther, and ten drops of the tinctura opii, in lieu of the effervescent draughts; and, when the appetite allowed it, I permitted soup, arrow-root, sago, or any other mild nourishment, to be taken in moderate quantities: but the stomach was in general so weak, as not to call for any thing more than what was given him in the way of medicine. My reason for ordering rum in preference to other stimulating liquor, is, that the patients who have been under my care have been accustomed to that spirit. To patients of a different sphere in life, I would allow wine or porter, as the circumstances might indicate. When constipation prevailed, I found a drop of the croton oil a most useful medicine; as, in addition to its efficiency as a purgative, it appears to act through the medium of the nervous system, and therefore becomes a desideratum in this stage of the disease. At this particular stage of the complaint, or that of exhaustion, I would not have recourse to large doses of opium, as is indiscriminately recommended by authors: my object at this period would be to raise the lowered scale of nervous power, not by overwhelming it with over-doses, but by the gradual effect of stimulants, and the aid of opium in quantities calculated to allay irritation without increasing debility. The means here mentioned should be persisted in, with modifications as the symptoms increase or diminish, and with a view to the general principles already laid down, which, if properly administered and attended to, will in many cases cut short the disease, by averting the second stage and inducing sleep; in the same way as the warm bath, in the cold stage of ague, may bring on diaphoresis, and thereby shorten the paroxysm. I

have witnessed this effect in several instances lately. If, however, after all our efforts, the second stage, or that of nervous re-action, should supervene, we must not be discouraged, but then act on the principles generally recommended,—namely, by the administration of full doses of opium; taking care at the same time to support the efforts of the system by the assistance of diffusible stimuli and antispasmodics, such as rum, brandy, wine, or porter, and camphor mixture with æther, as directed in the first stage, varying their administration according to circumstances. To these I have been in the habit of adding calomel and Dover's powder, (say two grains of the former and six of the latter every two hours,) until the system became affected or the disease yielded. Mercury may be of service here, from its deobstruent and equalising effects. The warm bath should also be prescribed with the same view, but particularly to soothe nervous irritation, and favour an equal distribution of the blood by exciting general perspiration; during the absence of which, cold applications ought to be constantly kept to the head, in order to diminish sensorial action. The state of the bowels ought to be watched; and, whether costiveness or the nature of the egesta render evacuations necesary, the croton oil becomes as essential a remedy in this as in the first stage. We must persist in this mode of treatment until a favourable change is foreseen, which will manifest itself by a diminution of all the symptoms, and a tendency to sleep. Care must be taken, at this particular approach of crisis, not to interfere too much with the intentions of nature, by over-doses of medicine, particularly of opium: and I must here mention, that I think, after a certain fair quantity of that active drug be administered, we ought to be cautious in giving more, without allowing sufficient time to intervene between the doses; for, as Dr. Pearson observes, "if they do not succeed at their first operation, they add much to the intellectual confusion, and are fraught with danger." It does not appear to me to be of any service to attempt to break the chain of morbid concatenation too abruptly, as the stage of mental irritation seems to require a given time to subside, in proportion to the duration of the stage of exhaustion, to the mode of treatment adopted, and to its previous causes.

From the beginning of this stage, our most particular attention ought to be directed to the moral management of the patient; and we must endeavour, by all the means in our power, to gain ascendency over his mind, without having recourse to coercive measures. In fact, the principles laid down by Pinel in the cases of mania become particularly applicable to this disease; but, as they are generally known, a recapitulation of them here is not necessary. Should sleep come on, it will be at first disturbed, and accompanied with nervous startings; it is, therefore, to be encouraged by all the means in our power: all noise should be avoided, and, in hot climates, a mosquito-net ought to be let down, so as to preclude any annoyance from insects. Should the patient awake soon, he will often be in a state of alarm and nervous agitation; but, if some warm stimulating drink, with a moderate dose of opium, be given, and mild and assuring conduct adopted, he will in every instance very soon fall into a profound sleep; from which, through the restorative effects of "nature's soft nurse," he generally awakes perfectly rational; after which we have little more to do than support the strength, and gradually diminish the quantities of stimuli which we have been in the habit of administering, so as to bring the constitution back to a healthy and moderate degree of excitement.

Should sleep not supervene in this disease, and untoward symptoms (such as have been described in the course of this paper,) ensue, we must persist in the general principle of our treatment, but with variation according to existing indications: thus, at this period, when effusion has either commenced or is about to take place, I would recommend, contrary to the general opinion, the application of a blister to the head, and the liberal administration of musk and ammonia. I would also blister the extremities, order mercurial frictions to excite the action of the absorbents, and continue the use of the warm bath, &c.; though, where the symptoms have attained this height, I fear little benefit can be derived from medical assistance, as it is more than probable effusion has taken place, which is generally the forerunner of death.

I have not taken any notice of blood-letting as a remedy in this disease, as I have seen but few cases requiring it: however, I do not say that it may not become a necessary, and even a very efficacious auxiliary, when practised in young and vigorous subjects, and particularly during the height of the second stage of the complaint; but it is on no account to be carried further than merely to relieve the temporary morbid excitement; to effect which the detraction of a few ounces of blood will be usually sufficient, and a repetition of it seldom necessary.

I have now terminated what I had to offer on the subject of this interesting disease, and, in concluding, I beg the indulgence of my reader for trespassing so much on his time with so ill-digested a composition; but, as it would be rather too much to expect that every army-surgeon should be endowed with the qualifications necessary to render him capable of being an author, and at the same time it would be culpable in him to withhold, on such grounds, the result of his experience in the pathology and treatment of an affection which is, as yet, not generally understood. I have chosen rather to expose myself to the tribunal of criticism, than to incur the stings of self-reproach.

TABLE *showing the Duration of the various Stages of the Cases of* DELIRIUM EBRIOSITATIS, *treated in the Regimental Hospital of the 5th Foot, in the Year* 1822.

| *Cases.* | *Age of the Patient.* | *Diseases for which admitted.* | *Date of Admission.* | *Date of the Commencement of Mental Derangement.* | *Date of the Commencement of the Sleeping, or third Stage.* | *Date of Death.* | *Remarks.* |
|---|---|---|---|---|---|---|---|
| First .... | 32 years | Dysent. Acut. .. | 5th June, 1822 | June 8th, 1822 | June 10th, 1822 | ...... | The fourth case was merely febrile when admitted, but was returned Delirium Tremens, by the desire of Mr. Doughty; though that affection did not come on until the third day after admission. |
| Second .. | 40 .. | Obstipatio ...... | 21st .. .. | .. 24th, .. | .. 27th, .. | ...... | |
| Third.... | 32 .. | Febris Cont. Com. | 9th August, .. | August 11th, .. | August 13th, .. | ...... | |
| Fourth .. | 32 .. | Delirium Tremens | 17th .. .. | .. 20th, .. | .. 22d, .. | ...... | |
| Fifth .... | 32 .. | Febris Cont. Com. | 1st Septemb. .. | Septem. 4th, .. | Septem. 6th, .. | ...... | |
| Sixth .... | 36 .. | Ambustio ........ | 12th .. .. | .. 15th, .. | .. 17th, .. | ...... | |
| Seventh .. | 40 .. | Cholera Morbus | 14th .. .. | .. 18th, .. | .. 21st, .. | ...... | |
| Eighth .. | 32 .. | Febris Cont. Com. | 25th .. .. | .. 27th, .. | .. 30th, .. | ...... | |
| Ninth .. | 31 .. | Idem ........... | 30th .. .. | October 5th, .. | October 8th, .. | ...... | |
| Tenth .. | 33 .. | Idem ........... | 3d Novemb. .. | Novem. 5th, .. | .... | Nov. 7th, 1822 | |

NOTE.—A greater number of cases of Delirium Tremens appear in this Table than in the General Return: this arose from my neglecting to change the name of the disease when it came on.

